# Potential Elimination of Human Gut Resistome by Exploiting the Benefits of Functional Foods

**DOI:** 10.3389/fmicb.2020.00050

**Published:** 2020-02-11

**Authors:** Christina Tsigalou, Theocharis Konstantinidis, Elisavet Stavropoulou, Eugenia E. Bezirtzoglou, Athanasios Tsakris

**Affiliations:** ^1^Laboratory of Microbiology, Medical School, University Hospital, Democritus University of Thrace, Alexandroupolis, Greece; ^2^Centre Hospitalier Universitaire Vaudois, Lausanne, Switzerland; ^3^Laboratory of Food Science and Technology, Department of Agricultural Development, Democritus University of Thrace, Orestiada, Greece; ^4^Department of Microbiology, Medical School, National and Kapodistrian University of Athens, Athens, Greece

**Keywords:** antimicrobial resistance, gut microbiome, resistome, antimicrobial resistance genes, probiotics, prebiotics

## Abstract

Recent advances in technology over the last decades have strived to elucidate the diverse and abundant ecosystem of the human microbiome. The intestinal microbiota represents a densely inhabited environment that offers a plethora of beneficial effects to the host’s wellbeing. On the other hand, it can serve as a potential reservoir of Multi-Drug Resistant (MDR) bacteria and their antibiotic-resistant genes (ARgenes), which comprise the “gut resistome.” ARgenes, like antibiotics, have been omnipresent in the environment for billions of years. In the context of the gut microbiome, these genes may conflate into exogenous MDR or emerge in commensals due to mutations or gene transfers. It is currently generally accepted that Antimicrobial Resistance (AMR) poses a serious threat to public health worldwide. It is of paramount importance that researchers focus on, amongst other parameters, elaborating strategies to manage the gut resistome, particularly focusing on the diminution of AMR. Potential interventions in the gut microbiome field by Fecal Microbiota Transplant (FMT) or functional foods are newly emerged candidates for the uprooting of MDR strains and restoring dysbiosis and resilience. Probiotic nutrition is thought to diminish gut colonization from pathobionts. Yet only a few studies have explored the effects of antibiotics use on the reservoir of AR genes and the demanding time for return to normal by gut microbiota-targeted strategies. Regular administration of probiotic bacteria has recently been linked to restoration of the gut ecosystem and decrease of the gut resistome and AR genes carriers. This review summarizes the latest information about the intestinal resistome and the intriguing methods of fighting against AMR through probiotic-based methods and gut microbial shifts that have been proposed. This study contains some key messages: (1) AMR currently poses a lethal threat to global health, and it is pivotal for the scientific community to do its utmost in fighting against it; (2) human gut microbiome research, within the last decade especially, seems to be preoccupied with the interface of numerous diseases and identifying a potential target for a variety of interventions; (3) the gut resistome, comprised of AR genesis, presents very early on in life and is prone to shifts due to the use of antibiotics or dietary supplements; and (4) future strategies involving functional foods seem promising for the battle against AMR through intestinal resistome diminution.

## Introduction

The human gut microbiota constitutes a whole world of microbes, fungi, viruses, and parasites in an eternal interplay between them and their host. This abundant and diverse ecosystem, with more than 100 trillion of microorganisms, has created a unique niche and plays a pivotal role in its host’s physiology and metabolism by participating in digestion, innate and adaptive immunity, and protection from pathogen colonization. During the last decade, culture-independent techniques have set out to elucidate details for the dominant phyla: the external and internal factors influencing the intestinal microbiome, its “normal” and “abnormal” variations, and its relationship with health and disease (Rinninella et al., 2019).

On the other hand, antimicrobial resistance (AMR) has managed to besmear the miraculous antibiotics and endanger the achievements of modern medicine by nullifying the ability of antimicrobials to fight infections. The misuse and overuse of antibiotics promote bacterial resistance, and the malleable gut microbiota has consequently evolved into a reservoir of resistance genes, even from infancy (Francino, 2016).

Apart from strategies that involve Antibiotic Stewardship – the implementation of policies and new drugs in order to re-establish the therapeutic capability of antibiotics – a very promising solution has emerged from the field of functional foods. It’s becoming more than obvious that the gut resistome might be receptive to probiotic and prebiotic supplementation. The preferable result of this manipulation would be the elimination or even the uprooting of Multi-Drug Resistant (MDR) strains and restoration of dysbiosis and gut resilience (Wu et al., 2016; MacPherson et al., 2018).

In this paper, we aimed to provide an overview for the emergence and features of the gut resistome and the dynamics of prebiotics and probiotics administration targeting antibiotic-resistant genes (AR genes) as an alternative to tackling AMR today in the context of personalized medicine.

## The Gut Microbiome in a Nutshell

Nowadays, it is generally accepted that microbial cells on and in our body outnumber our human cells, and the most densely populated site is the Gastrointestinal Tract (GI), which presents a gradual augmentation from 10^4^/ml bacterial cells in the stomach and duodenum to almost 10^11^/ml in the colon (Sender et al., 2016). Either a “virtual organ” (Valdes et al., 2018) or a multiple set of “organs” (Cani, 2017) has become, in the last two decades, a very intriguing area of research, especially following the introduction of new technologies for analyzing DNA and RNA (16S rRNA sequencing–Shotgun metagenomics sequencing) directly from feces. The dominant phyla *Firmicutes* and *Bacteroidetes* represent 90% of gut microbiota (Arumugam et al., 2011), and Europeans are categorized to three enterotypes according to its variations (Zoetendal et al., 2008; Segata et al., 2012; Stavropoulou et al., 2019).

In contrast to previous beliefs that newborns were free of bacteria, it is now a proven fact that bacteria are present in the endometrium, amniotic fluid, umbilical cord, and meconium (Amenyogbe et al., 2017), and different environmental factors, such as the delivery mode (cesarean or vaginal), feeding style (breastfeeding or formula), gestational age, administration of probiotics or prebiotics, diet, exercise, geography, genetics, and air pollution are the most pivotal determinants for gut microbiota modifications that lead to the development of a mature and healthy gut (Bezirtzoglou and Stavropoulou, 2011; Koren et al., 2013; Tanaka and Nakayama, 2017; Thursby and Juge, 2017; Angelakis and Raoult, 2018; Avelar Rodriguez et al., 2018; Ficara et al., 2018; Leoni et al., 2019). It’s quite controversial whether the human placenta includes microbes. Aagaard et al. (2014) studied placental specimens collected under sterile conditions from 320 subjects. Authors have characterized a unique placental microbiome niche, composed of non-pathogenic commensal microbiota from the *Firmicutes, Tenericutes, Proteobacteria, Bacteroidetes*, and *Fusobacteria* phyla. The placental microbiome was closely related to the human oral microbiome (Bray–Curtis dissimilarity <0.3) (Aagaard et al., 2014). On the other hand, de Goffau et al. (2019) as well as McDonald and McCoy (2019) in their respective studies, concluded that the human placenta is microbe-free. Studies have demonstrated that diversity and richness increases during life until adulthood when the intestinal microbiota stabilizes and, later on, reduces and adopts a poorer phenotype until the onset of old age (Belizário and Napolitano, 2015). A mutually beneficial relationship is established between the gut microflora and human host. This is called “eubiosis,” and, among other things, it aids in regulating digestion, immune function, resistance against pathogens, and the maintenance of an intact alimentary track epithelium (Brestoff and Artis, 2013; Avelar Rodriguez et al., 2018). Any disruption of this balance leads to changes in composition and diversity, called “dysbiosis,” and this has a negative impact on host health. Although the causality of the microbiome shifts has not yet been fully proven, a plethora of studies implicate this imbalance in multiple intestinal and extra-intestinal diseases, namely obesity, autoimmunity, cancer, allergies, neurological disorders, autism, etc. (Gao et al., 2017; Tanaka and Nakayama, 2017; Castaner et al., 2018; Clemente et al., 2018).

The restoration of dysbiosis by different kinds of approaches, such as neutraceuticals, diet interventions, and FMT, is a whole new scientific field, and, though poorly understood, it is very compelling and full of promise.

## Antimicrobial Resistance and the Rise of Another –OME: the Resistome

Even though the emergence of antibiotics in the 1930s signified a new era in fighting infections, the truth is that they have been present in nature for eons. Unfortunately, their widespread usage has contributed to the growing global threat of AMR, which has resulted in the reduction of therapeutic options as well as a rise in lethal infections from MDR or PDR (Pan Drug Resistant) bacteria and “superbugs” in healthcare settings worldwide.

Bacteria have dwelled on the Earth for ages and have evolved the capability to avoid antibiotic attack through an accumulation of mutations or by a scattering of AR genes, using methods including horizontal gene transfer (transduction, conjugation, and transformation), toxin–antitoxin systems, and Mobile Genetic Elements (MGEs) (transposons, plasmids, integrons, and integrative–conjugative elements) (Burrus et al., 2002; Carattoli, 2013; Iyer et al., 2013; Sultan et al., 2018; Xie et al., 2018).

Antibiotic-resistant genes are mostly harbored by strictly anaerobic commensals in the gut, but, when a transfer of genetic material occurs by chance to pathobionts, it is conferred to the proliferation of MDR strains (van Schaik, 2015). Collectively all the AR genes and their precursors of gut bacteria pathogens and non-pathogens make up the resistome, as proposed by Gerard D. Wright in 2006 and 2007 (D’Costa et al., 2006; Wright, 2007). The intestine is considered a huge reservoir for antibiotic resistance determinants. The preponderance of determinants can, however, be considered inherent, and they are rarely shared with opportunistic pathogens (Ruppé et al., 2019). This is possibly due to the presence of unknown obstacles for this transfer from gut anaerobic eubionts (mainly *Bacteroides*) to Gram-negative facultative anaerobic pathobionts (e.g., Enterobacteriaceae) (van Schaik, 2015). Less is known concerning the role of Firmicutes in this procedure. Furthermore, the MGEs form the “mobilome” as a vehicle of transferring AR genes amongst gut microbes (Frost et al., 2005; Ravi et al., 2015, 2017; Pärnänen et al., 2018).

Over the last decades, opportunistic pathogens, such as *Escherichia coli, Klebsiella pneumoniae, Enterococcus faecalis*, and *Enterococcus faecium* from the *Enterobacteriaceae* and *Enterococcaceae* groups, represent very important MDR strains in hospitals that may cause serious infections when they translocate across the gut barrier. From the 1970s and 1980s, microbial “culturomics” have shown that Bacteroidales and Clostridiales may bear and transfer AR genes, not only among themselves but to pathobionts as well (Guiney and Davis, 1978; Privitera et al., 1979; Wüst and Hardegger, 1983; van Schaik, 2015). Metagenomic research suggested that effective antibiotic stewardship as a “One Health” policy (humans, animals, and the environment) may contribute to great changes regarding the abundance of AR genes in the gut microbiome of different countries’ dwellers and that resistance genes show specific geography clusters (Forslund et al., 2013; Hu et al., 2013). Likewise, hospitalized patients comprise a particular population under intense and (usually) prolonged antibiotics therapy; metagenomics revealed an increase in relative abundance of resistance genes, such as aminoglycosides (de Smet et al., 2009; Buelow et al., 2014).

Several recent studies have confirmed that the adult intestine has lower richness in AR genes than that of an infant, even if there is not prior exposure to antibiotics (Bäckhed et al., 2015; Moore et al., 2015; Gibson et al., 2016). There is conflicting evidence for the origins of these resistance genes, but most of it derives from mother’s gut as well as breast milk microbes (Zhang et al., 2011; Bode, 2012; Jost et al., 2014; Gosalbes et al., 2016). In this context, there is a hypothesis that this high percentage of AR genes, especially in preterm infants, originates from the high levels of Gammaproteobacteria among first commensals, which usually carry resistance genes (Hu et al., 2013; Moore et al., 2015; Gosalbes et al., 2016). Breastfeeding, apart from numerous other benefits, seems to suppress Gammaprotobacteria and promote Bifidobacteria, which are negatively related to resistance and consequently eliminate the antibiotics resistance burden of the infant gut, especially during the first 6 months (Bode, 2012; Moore et al., 2013; Moore et al., 2015; Pärnänen et al., 2018).

The impact of antibiotics upon the gut microbiota as a cause or promotor of dysbiosis has been widely investigated, and specific features, such as dosage and duration, have been implicated in the elimination of certain commensals that offer colonization resistance. Li et al. (2019) observed that even short-term antibiotic administration led to an individualized resistome shift and altered predominance of certain strains and their personal dynamics, thus indicating a new era in personalized therapy. The most investigated example is the *Clostridium difficile* as the cause of types of nosocomial diarrhea due to extended medication with broad-spectrum antibiotics (mainly clindamycin) (Casals-Pascual et al., 2018). The latest recommended therapy for recurrent *C. difficile* infection is FMT (Buffie and Pamer, 2013; Pamer, 2016). Moreover, there are studies showing the elimination of AR genes and MDR bacteria, indicating the potential use of the method for restoring colonization resistance in the gut, but this requires more elaboration (Francino, 2016; Marchesi et al., 2016). It is worth mentioning that the temporary use of probiotics (well-characterized beneficial gut microbes) in tandem with antibiotics therapy is correlated with lower risk of developing clinical overt *C. difficile* infection (Goldenberg et al., 2018).

**FIGURE 1:**
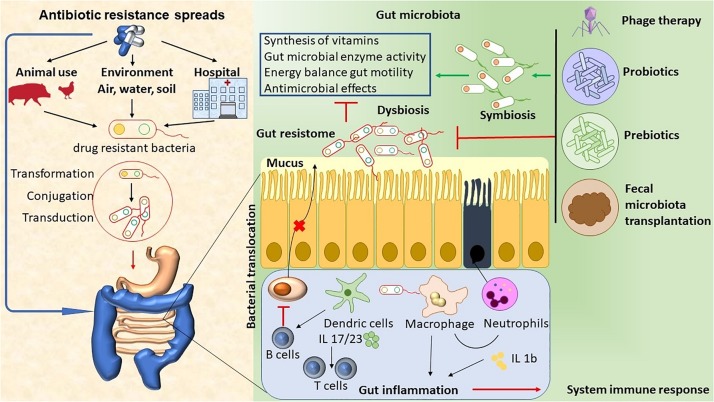
The abuse of antimicrobial agents (environmental, hospital, or animal) has lead to disturbances in the ecological community of the gut microbiota. This leads to mucus layer dysfunction and increased bacterial translocation. These bacteria promote the activation of DCs and macrophages by microorganism- associated molecular patterns (MAMPs) through Toll-like receptors (TLRs), thus inducing neutrophil chemotaxis and IL production. Local inflammation leads to epithelial cells damage. APC present antigens to prime and maintain T-cell responses, and B cells inhibit the production of Ig A by plasma cells. Finally, immune system activation promotes inflammation, which ultimately leads to chronic diseases. The therapeutic approaches, such as phage therapy, the use of functional foods with probiotics and prebiotics, and fecal microbiota transplant (FMT), have the potential to target specific bacterial taxa, thus helping to re-establish homeostasis and microbiome balance.

The abuse of antimicrobial agents (environmental, hospital, or animal) has lead to disturbances in the ecological community of the gut microbiota. This leads to mucus layer dysfunction and increased bacterial translocation. These bacteria promote the activation of DCs and macrophages by microorganism-associated molecular patterns (MAMPs) through Toll-like receptors (TLRs), and they induce neutrophil chemotaxis and interleukin (IL) production (Luca and Shoenfeld, 2019). Local inflammation can lead to damage to the epithelial cells. Antigen-presenting cells (APC) present antigens to prime and maintain T-cell responses, and B cells inhibit the production of IgA by plasma cells. Finally, the immune system’s activation promotes inflammation, which ultimately leads to an array of chronic diseases (Guven-Maiorov et al., 2017; Luca and Shoenfeld, 2019). Therapeutic approaches, like phage therapy, the use of functional foods with probiotics and prebiotics, and fecal microbiota transplantation (FMT), have the potential to target specific bacterial taxa, thus helping to re-establish homeostasis and a microbiome balance (Figure 1).

## Functional Foods and Gut Microbiota Modifications

The gut microbiota ecosystem, in order to keep its role in the interface of inner and outer worlds, requires the security of the equilibrium state of eubiosis-homeostasis. The rather stable adult intestinal microbiome is characterized by resilience and plasticity, meaning that, after dietary changes, antibiotics, or infections, it has the ability to mostly restore its shifts in diversity and abundance within days or months (Turnbaugh et al., 2007; Lozupone et al., 2012). Several interventions for resurrecting the lost “healthy” gut microbiome encompass bacteriotherapy (by FMT), prebiotics (compounds that benefit microbes), and probiotics (certain beneficial gut strains) (Al-Kharousi et al., 2018).

Functional foods usually acquire additional functions (often related to health promotion or disease prevention) by adding new ingredients or more of the existing ingredients (Functional Foods and Nutraceuticals, 2008), ingredients like probiotics and prebiotics amongst others. The World Health Organization defines probiotics as “live microorganisms which when administered in adequate amounts confer a health benefit on the host” (Food and Agriculture Organization of the United Nations, World Health Organization, 2006), and prebiotics are non-digestible food components that promote the growth and vivacity of commensals (Shafi et al., 2014). Prebiotics have the capacity to upgrade the expansion and metabolic activities of probiotics. Therapeutic synbiotics serve as a nutritional supplement, referred to as “combination of prebiotics and probiotics in the form of synergism that advantageously affect the host” (Gibson and Roberfroid, 1995; Bezirtzoglou and Stavropoulou, 2011).

Probiotic strains present antimicrobial activity, and they inhibit the growth and displace the adhesion of potential pathogens to human mucus. Collado et al. (2007), tested different probiotic strains against eight selected pathogens. The results indicated that all probiotic strains tested were able to inhibit and displace the adhesion of *Bacteroides, Clostridium, Staphylococcus*, and *Enterobacter* (Collado et al., 2007). Furthermore, probiotics restore mucosal homeostasis (Suchodolski and Jergens, 2016), and they also play a pivotal role in the prevention and treatment of *H. pylori* (Gotteland and Cruchet, 2003) and have a positive effect upon allergic conditions (Forsythe et al., 2007) etc. Prebiotics mainly yield a food source for the flourishing of probiotics and also increase glucose uptake (Breves et al., 2001) and the minerals’ bioavailability (Smiricky-Tjardes et al., 2003).

The exact short- and long-term effects of probiotics and prebiotics on intestinal microbiota are not yet fully elucidated, and conflicting evidence exist. Their impact does not fit into a “one-size-fits-all” model (Sanz et al., 2018) and seems to be unique for every individual for any period of their life.

## Resistome Diminution Due to Dietary Supplementation – Is It Feasible?

Excessive antibiotics exposure, especially from early life or even in the uterus, leads to dysbiosis with numerous – yet not completely understood – consequences. Imbalances and disruptions of the intestinal microbiota stemming from antibiotics may decrease its efficiency in fighting infections, threaten immune homeostasis, decontrol metabolism, and gather AR genes that are capable of spreading across species (Francino, 2016). In this vein, the use of probiotic bacteria seems to be a very promising intervention either for preventing perturbations by synchronous administration with antibiotics or by tapping into their benefits for healing the “injured” microbiota and counteract the detrimental effects of antibiotics afterward.

But, as AMR currently addresses public health globally, efforts have been made, apart from the discovery of new drugs, to investigate alternative strategies to combat it. As the pipeline of research into antimicrobial agent, with a few exceptions, seems to dry up, dietary interventions are under active consideration for the time being. In Wu et al. (2016) showed in their study that a gut microbiota-targeted dietary intervention in obese children managed not only to alter the composition and functionality of the intestinal microbiome but, furthermore, to remarkably decrease their gut resistome. The diet that they had previously implemented (Zhang et al., 2015) included whole grains, traditional Chinese medicinal foods, and prebiotics (WTP diet), and with a genome-centric metagenomic approach, this resulted in a reduction of body weight and inflammation parameters. According to their data, other than weight loss and health improvements, they reported a decrease of the resistome richness, a reduction in the AR gene members, and the diminution of multiple-resistance AR gene carriers (reduced 69%), showcasing a possible novel perspective for the issue (Wu et al., 2016). Another important notion in this study is that, because of the diet type (high carbohydrate), Bifidobacterium was augmented and had few or no ARgenes, whereas the decreased bacteria resembled a minefield with their great burden combined with the final reduction of the resistome.

Researchers also demonstrated last year that MDR pathogens have different behaviors depending on the presence in their environment of co-pathogens, commensals, interactions with the host, and probiotics (Chan et al., 2018). Moreover, shifts in the gut microbiome and resistome after antibiotic administration succeeded in recovery within a week of the end of treatment, and this was probably because of the resilience and plasticity of the gut ecosystem, which acts in a very characteristic and predictable way (MacPherson et al., 2018). Evans et al. (2016) first introduced the presented evidence that *L. helveticus* R0052 and *L. rhamnosus* R0011 supplementation had a meaningful negative impact on the duration of diarrhea-like defecations following antibiotics.

Another target group that is relatively vulnerable to factors attributed to dysbiosis are infants, particularly preterm ones. As their microbiome is unstable and less diverse, probiotics administration may promote colonization resistance, protection from serious conditions such as necrotizing enterocolitis, and mitigate the negative impact of antibiotics (Esaiassen et al., 2018). Limitations to the procedures have to do with sampling, the duration of the probiotic supplementation, small number of participants, etc., but it is a very promising intervention for dealing with AMR nowadays.

## Breastfeeding in Early Life and Gut Microbiota

The gut microbiome is established during the newborn period. Different factors during prenatal, perinatal, and postnatal life may affect the gut microbial composition (Chong et al., 2018). Breastfeeding in early life configurates the gut microbiota, both directly by exposure of the neonate to the milk microbiota and indirectly via maternal milk factors, such as milk oligosaccharides, secretory IgA, and anti-microbial peptides that affect bacterial growth (van den Elsen et al., 2019). On the other hand, the infant gut microbiota has a high abundance of ARgenes, even in the absence of antibiotics exposure. Pärnänen et al. (2018), studied potential sources of infant gut ARgenes by performing metagenomic sequencing of breast milk as well as infant and maternal gut microbiomes. The results of this study suggested that fecal ARgenes of infants resembled those of their own mothers. The infants inherit the legacy of the past antibiotic consumption of their mothers via the transmission of genes (Pärnänen et al., 2018).

## Conclusion and Future Perspectives

The emergence of AMR jeopardizes all the achievements of medical science, and it is of paramount importance to urgently raise awareness of this fact and promote the need for the implementation of effective strategies. In this notion, the gut resistome represents a continuous menace for the host’s wellbeing and public health. Different approaches strive to eliminate it, and, amongst other proposed methods, the exploitation of functional foods is strongly considered. It is indeed a very intriguing and promising intervention, though little is yet known with regards to the real impact it can have upon the ARgenes and the potential diminution and restoration of dysbiosis. Further research and more detailed and personal data are essential in order to evaluate and standardize manipulations and functionalities of diet components in a more personalized approach.

## Author Contributions

All authors listed have made a substantial, direct and intellectual contribution to the work, and approved it for publication.

## Conflict of Interest

The authors declare that the research was conducted in the absence of any commercial or financial relationships that could be construed as a potential conflict of interest.
